# A Pilot Study on the Whole Exome Sequencing of Prostate Cancer in the Indian Phenotype Reveals Distinct Polymorphisms

**DOI:** 10.3389/fgene.2020.00874

**Published:** 2020-08-25

**Authors:** Ayam Gupta, Nidhi Shukla, Mamta Nehra, Sonal Gupta, Babita Malik, Ashwani Kumar Mishra, Maneesh Vijay, Jyotsna Batra, Nirmal Kumar Lohiya, Devendra Sharma, Prashanth Suravajhala

**Affiliations:** ^1^Department of Biotechnology and Bioinformatics, Birla Institute of Scientific Research, Jaipur, India; ^2^Vignan’s Foundation for Science, Technology & Research (Deemed to be University), Guntur, India; ^3^Department of Chemistry, School of Basic Sciences, Manipal University Jaipur, Jaipur, India; ^4^DNA Xperts Private Limited, Noida, India; ^5^Rukmani Birla Hospitals, Jaipur, India; ^6^Australian Prostate Cancer Research Centre, Queensland Institute of Health and Biomedical Innovation and School of Biomedical Science, Queensland University of Technology, Brisbane, QLD, Australia; ^7^Department of Zoology, University of Rajasthan, Jaipur, India

**Keywords:** prostate cancer, genomics, exome sequencing, prognosis, biomarkers

## Abstract

Prostate cancer (PCa) is the third most common cancer among men in India, and no next-generation sequencing (NGS) studies have been attempted earlier. Recent advances in NGS have heralded the discovery of biomarkers from Caucasian/European and Chinese ancestry, but not much is known about the Indian phenotype/variant of PCa. In a pilot study using the whole exome sequencing of benign/PCa patients, we identified characteristic mutations specific to the Indian sub-population. We observed a large number of mutations in DNA repair genes, *viz.* helicases, TP53, and BRCA besides the variants of unknown significance with a possibly damaging rare variant (rs730881069/chr19:55154172C/TR136Q) in the TNNI3 gene that has been previously reported as a semi-conservative amino acid substitution. Our pilot study attempts to bring an understanding of PCa prognosis and recurrence for the Indian phenotype.

## Introduction

Prostate cancer (PCa) is the second most prevalent cancer worldwide and the third most prevalent cancer in India (Jain et al., 2014). Over 1 million PCa cases are diagnosed per year globally, and the mortality rate has grown to more than 300,000 deaths per year (Farhat et al., 2000). The Population Based Cancer Registries (PBCRs) of different cities for the time period shows that PCa has ranked among the top leading sites of cancer in many cities in India (NCRP, 2013). Prostate cancer leads in large Indian cities like Delhi, Kolkata, Pune, and Thiruvananthapuram after oral cancer, and is the third leading site of cancer in cities like Bangalore and Mumbai besides being among the top 10 leading sites of cancers in the rest of the PBCRs of India (Jain et al., 2014). The data show that almost all regions of India are equally affected, with the incidences of PCa relatively low in some states like Gujarat (Ahmedabad and Wardha PBCRs) and Madhya Pradesh (Bhopal PBCR), the lowest being the northeast regions of India (Jain et al., 2014). This could be because of lack of PBCRs in addition to PCa not actively reported in states where awareness about it is lacking (Kimura, 2012).

Although studies on PCa have dealt with the genetics, genomics, and environmental influence in the causality of PCa, no association of genotype and phenotype employing the whole exome sequencing (WES) of PCa has been done to date for the Indian population. As many Indians are vegetarian, a polygenic risk with other diseases such as diabetes could be associated with PCa, which would be interesting to deliberate (Agrawal et al., 2014). On the other hand, studies at the sequence level in India have been limited where the genetic variants have been shown to be associated with PCa but not linked to pathogenesis (Abate-Shen and Shen, 2000; Shen and Abate-Shen, 2010). In the recent past, the next-generation sequencing (NGS) approaches have provided an efficient, rapid, economical, and global biological means to understand millions of sequenced DNA reads besides recognizing ample variants from the samples (Mardis, 2008). It has an increasing potential to carry a wide spectrum of purposes including many research fields, for example, in the molecular diagnostics of genetic diseases, infectious diseases, cancer, and pharmacogenomics (Ten Bosch and Grody, 2008; Guchelaar et al., 2014). On the other hand, the integration of systems biology with genomic data can lead to the profound knowledge of the disease pathway besides allowing us to identify prognostic biomarkers for drug target identification.

In this pilot study, while we report mutations from northwestern Indian patients, we aimed to understand the marked impact of the risk of PCa specific genes on affected individuals versus controls. We claim that this is perhaps the first study of its kind, where WES was employed to screen mutations and infer the genetic evaluation of PCa in India. With the new Genome India Project initiative (GenomeIndia Initiative, 2017), the genetic makeup for such diseases could allow us to understand the pathways associated with PCa native to India. We also deliberate on mutations associated with polygenic diseases and vitamin D deficiency, besides those that are unique to human diet, socioeconomic imbalances, and PCa manifestation when compared to the Caucasian population (Layne et al., 2019).

## Materials and Methods

### Subjects

The samples were collected from Rukmani Birla Hospital (RBH), Jaipur, and all patients were identified as native to north or northwest India. All methods were performed in accordance with relevant guidelines and regulations. Archival pathological FFPE blocks containing specimens were obtained retrospectively after clearance from the institutional ethics committee of RBH, and informed consent wherever applicable was duly taken. Annotated FFPE blocks of five benign and five tumors (malignant) were taken and sent to Xcelris Labs, Ahmedabad, for WES (Table 1). DNA from sample tissues was extracted using the FFPE tissue gDNA isolation kit (Qiagen/56404) for DNA isolation, and quality control (QC) for contamination was done. The baits for exon capture with a mean target coverage depth of 110× per sample were achieved with as many as several targets covered for more than 20×.

**TABLE 1:** Samples used for the WES along with their Gleason scores which constituted one high-grade tumor sample, with three intermediate samples and one just on par above grade 6, while five others are with less than six from benign cases.

Sample ID	Condition	Gleason Score (primary + secondary)
Z785	Adenocarcinoma	3 + 4 = 7
Z786	Acinar Adenocarcinoma	3 + 4 = 7
Z789	Acinar Adenocarcinoma	3 + 3 = 6
Z791	Acinar Adenocarcinoma	3 + 4 = 7
Z794	Cribriform Adenocarcinoma	4 + 4 = 8
Z787	Benign Nodular Prostatic Hyperplasia	<6
Z788	Benign Prostatic Hyperplasia	<6
Z790	Benign Nodular Prostatic Hyperplasia	<6
Z792	Benign Nodular Prostatic Hyperplasia	<6
Z793	Benign Nodular Prostatic Hyperplasia	<6

### Sample Preparation, Exome Capture, and Sequencing

DNA was isolated from samples using the Qiagen FFPE DNA extraction kit. The quality of genomic DNA was checked on 0.8% agarose gel (loaded 3 μl) for the single intact band. The gel was run at 110 V for 30 min; 1 μl of each sample was used for determining the concentration using the Qubit 2.0 Fluorometer. All the libraries were prepared using the Agilent SureSelect XT target enrichment system by following the manufacturer’s instruction. Briefly, 200 ng of each DNA sample was used for fragmentation using the Covaris S2 system. While the fragmented DNA was subjected to end repair, A-tailing, it was followed by adapter ligation which was hybridized to RNA baits that are designed to physically capture specific DNA sequences. The captured DNA is then eluted from the baits, purified using a biotin-based precipitation, and then amplified by PCR to yield an exome library for sequencing. All prepared libraries were checked on the Agilent high-sensitivity (HS) chip on Bioanalyzer 2100 and quantified on fluorometer by the Qubit dsDNA HS Assay kit (Life Technologies). The average size and concentration of each library was calculated from the HS chip and Qubit, respectively (see Table 2).

**TABLE 2:** Data statistics of the sequenced samples.

File Name	Total Reads	Total Bases		Size in GB
Z785	34,589,627	4,524,820,258	9,035,044,752	9.03
Z785	34,589,627	4,510,224,494		
Z786	36,192,378	4,782,261,247	9,552,530,147	9.55
Z786	36,192,378	4,770,268,900		
Z787	29,342,454	3,504,276,014	7,023,683,778	7.02
Z787	29,342,454	3,519,407,764		
Z788	32,007,925	4,590,702,434	9,142,339,304	9.14
Z788	32,007,925	4,551,636,870		
Z789	32,912,966	4,688,597,419	9,332,868,751	9.33
Z789	32,912,966	4,644,271,332		
Z790	44,212,484	6,398,843,741	12,730,582,043	12.73
Z790	44,212,484	6,331,738,302		
Z791	33,311,709	4,822,305,019	9,600,461,768	9.60
Z791	33,311,709	4,778,156,749		
Z792	36,245,502	5,367,958,348	10,658,814,541	10.65
Z792	36,245,502	5,290,856,193		
Z793	37,261,051	5,344,729,573	10,640,702,373	10.64
Z793	37,261,051	5,295,972,800		
Z794	33,200,697	4,584,192,242	9,148,425,756	9.14
Z794	33,200,697	4,564,233,514		

The amplified library was analyzed on Bioanalyzer 2100 (Agilent Technologies) using a HS DNA chip as per the manufacturer’s instructions. After obtaining the Qubit concentration for the library and mean peak size from Bioanalyzer, the profile library was loaded onto the Illumina platform for cluster generation and sequencing. Paired-end (PE) sequencing allowing the template fragments to be sequenced in both forward and reverse directions was ensured with the library molecules bound to complementary adapter oligos on PE flow cells. The adapters were designed to allow the selective cleavage of the forward strands after the re-synthesis of the reverse strand during sequencing. Finally, the copied reverse strand was used to sequence from the opposite end of the fragment, and all the libraries were sequenced with 150 × 2 PE reads, which resulted in 8–10 GB of data per sample.

### Quality Control and Variant Calling

**FIGURE 1:**
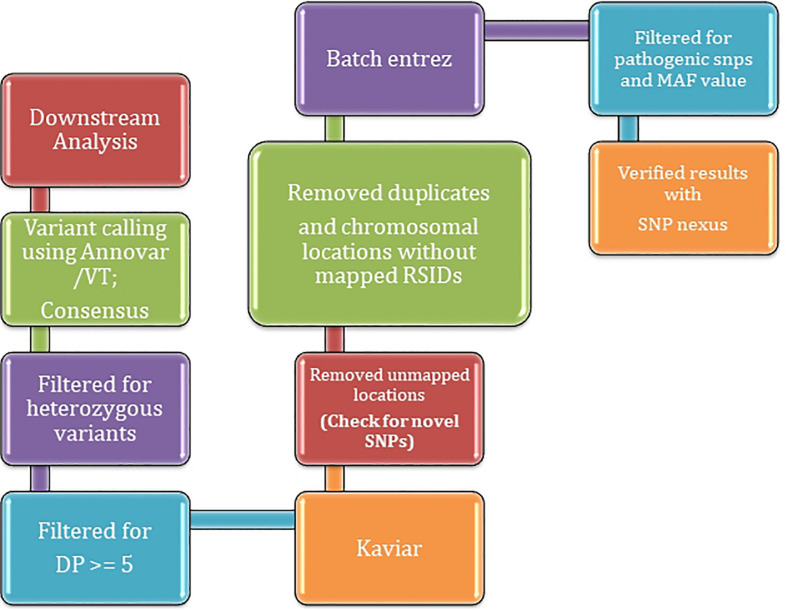
A brief overview of methodology employed for inferring variants using WES.

The datasets were run through our in-house pipeline described earlier (Meena et al., 2018). The quality assessment was done for all the samples using FastQC (Andrews, 2010) with raw reads checked for quality, GC bias, K-mer quality, and duplication levels. Calling and filtering of variants and indels were performed using Vt (Tan et al., 2015) and Annovar (Wang et al., 2010) before we established the sensitivity and specificity of called variants. Mutations were counted as heterozygous (“het”) using awk/bash one-liners and the two sets of average depth (≥5 DP ≤ 20 and DP ≥ 20) were parsed for further downstream annotation (Supplementary Information). Population stratification at a gross-level was deemed not necessary given the small size of the population on a small scale; however, contamination crosscheck both at sequence (fastq and bam levels) were ensured to assess the heterogeneity of each given site of pooled dataset. Variant analysis using Vt predicted the variant types and were filtered by setting criteria for a false discovery rate (FDR) that yielded the genomic variants in affected individuals versus controls. The samples were analyzed for mutations predicted as causal, and the corresponding genes were filtered against GeneMania (Warde-Farley et al., 2010) and checked for the significant association of pathogenesis with the risk of genitourinary/PCa-specific genes. A brief overview of the methodology is described in Figure 1.

### Downstream Analyses

Downstream annotation was done using various open source tools. The reported PCa variants were confirmed from NCBIs’ ClinVar (NCBI ClinVar Database, 2019) using the search term “prostate” with filters “pathogenic” and “single nucleotide” and checked against the generated VCF files. The bcftools was employed to retrieve the heterozygous mutations with the varied depth as mentioned earlier. A final list of variants was validated using Sanger sequencing. For the latter, we have maintained an average of 400–600 bp of product size with the 200–250 bp flanking from both sides of the location of the SNP mutation. Primers were designed in such a way that secondary structures in the genome were avoided. We adjusted the GC content as 50% to maintain a balanced distribution of GC-rich in CDS regions. The PCR was set with initial denaturation at 95°C for 5 min, followed by 35 cycles of denaturation at 95°C for 30 s, annealing at 60°C for 30 s and an extension at 72°C for 45 s and final extension of 5 min at 72°C.

## Results and Discussion

### Characteristics of PCa Relevant to the Indian Phenotype

Ten subjects, including five cases, those including one high Gleason grade sample (8), and four between 6 and 7 grades were subjected to WES. The sequences were aligned to human genome reference (build hg38) using bowtie2 to produce the sequence alignment file. We observed 944,509 annotated variants and upon further normalization, we discovered 897,926 SNPs, indels and a few copy number variants (CNVs) among them. Post downstream analyses, the generated VCF files were filtered for the variants which were heterozygous and 42 SNPs were confirmed that exhibited significant association with PCa in affected subjects (Supplementary Table 1). Among them, 17 SNPs were subjected further to Sanger validation in an independent platform wherein primers were designed with a length of 18–25 nucleotides (Supplementary Table 2). We found that all the SNPs/samples except one unaffected (rs73598374) passed the sequencing quality thresholds from quality checks with a mean read depth of 14.4. The false positives were carefully checked between benign prostate hyperplasia (BPH) and PCa samples even as a number of exome sites, low-coverage sites, and large deletions could not be validated. In total, we observed 671,609 heterozygous variants confirming the above thresholds that were filtered across all samples.

### Association of Variants With PCa

**FIGURE 2:**
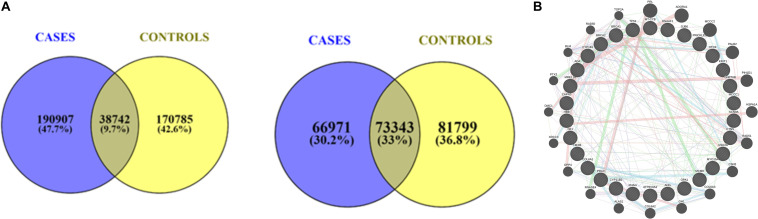
**(A)** A union of intersection of variants between cases and controls with respect to depth ≥ 5 DP ≤ 20 respectively. **(B)** Protein-Protein Interaction (PPI) map of genes associated with DNA repair genes.

The variants segregating from cases were retained, while those from controls were excluded to rule out the false positives. The minor allele frequency cut-off 0.05 intersecting the allele frequency were judiciously checked for low penetrated variants that are associated with a large number of disease susceptibility genes, primarily cancers. Therefore, we setup the variant identification to as low as DP = 5 and further, segregated the variants those that fall in ≥ 5 DP ≤ 20 and DP ≥ 20 (Figure 2A).

### High-Impact Variants in High-Grade Tumors

The identification of tumor-specific somatic mutations was possible with our pipeline. Among the inferred mutations, the prominent were BRCA2 mutations (rs276174889, rs80358600, rs80359171, rs771203198, and rs145988146) associated with high-grade and intermediate tumors (Z794, Z785, and Z786) (Couch et al., 2007). While, BRCA gene mutations lead to development of breast and ovarian cancer, they have been linked to PCa in addition to pancreatic cancer and other myelomas (Paul and Paul, 2014; Cavanagh and Rogers, 2015). The BRCA2 mutations in particular have been associated with an 8.6-fold increased risk for the PCa manifestation (Castro and Eeles, 2012) even as BRCA1 was also seen characteristically in one sample (Z785): rs28897696. Common genetic alterations are usually associated with heterozygous BRCA1 or BRCA2 mutations, and these include loss of the wild-type BRCA1 or BRCA2 allele (LOH), loss of TP53 (which encodes p53), and ATM or CHK2 function (Roy et al., 2012). These additional alterations may allow cells to bypass checkpoint controls and evade apoptosis, and thereby initiate tumorigenesis. Additionally, tumor complexity makes the detection of cancer-specific CNVs even more difficult (Zare et al., 2017), and the variants observed are frameshift, loss, or gain of stop codons which have significant insertions/deletions at a number of bases in different reading frames with altered protein sequences. While we found them to be significantly associated, we observed that mutations in TP53 and COL6A1 among the other 102 frameshift variants were shown to be affected with spliced regions. Sanger validation (orthogonal) was used to disprove a false-positive variant wherein we performed a systematic validation of variants of these genes and found no discrepancies among them.

The majority of mutations that are detected in the current study (*viz., GJB6, KRIT1, GNPTAB, ANG, MCM8*, and *NF1*) are only significant to high-grade PCa. The SNPs have been widely studied in context to cancer genomes and its predisposition, and so, we believe these SNPs cannot be associated with the familial aspect of the cancer. On the other hand, we observed that a few pathogenic SNPs were absent in high- and median-grade PCa samples and present in the low-grade sample, *viz., BRAF, RAD51C, RHAG, CYP11B2, PRICKLE1, CAPN3.* With the CNVs considered one of the most important somatic aberrations in cancer since oncogene activation, it is often attributed to chromosomal copy number amplification and tumor suppressor gene inactivation leading to largely heterozygous deletion. With the identification of somatic CNVs known to have an important role in cancer prognosis and treatment, we asked if any of them were significantly associated. On the other hand, we also found 76 CNVs associated with the gain/loss of function that we mapped using SNP nexus (Dayem Ullah et al., 2018) (Supplementary Table 3).

As expected, a large number of DNA repair genes (e.g., helicase, TP53, BRCA2) harbor characteristic mutations, which is in agreement with the WES studies. We also found possibly damaging rare variants of unknown significance (VUS) (rs730881069/chr19:55154172: C/T: R136Q) in the *TNNI3* gene that has been previously reported as a semi-conservative amino acid substitution. The mutation, howev2wer, is known to impact the PCa cases and seen across multiple hypertrophic cardiomyopathy case reports and, hence, classified as likely pathogenic. From the pathway and interaction network analyses, we inferred that *BRCA2* and *BRCA1* have a majority of interacting partners that are largely associated with either DNA repair genes or helicases, *viz., KRIT1, CTNS, USH2A, HEXB, MCCC1, MYO15A, CAPN3, GNPTAB, RHAG, PRLR, OTOF, GJB6, MCM8, CYP1B1, PRICKLE1, TP53, COL6A1, DNAAF1, SCN9A, CYP11B2, ATP6V0A2, NF1, ADA, MT-CYB, OPA1, ANG, HBB, MRE11* (Figure 2B). Notable among them is RecQ/BLM helicase, which has been recently reported to regulate cancer cell proliferation (Qian et al., 2017). In the recent past, recommendations demonstrated the role of prostate serum antigen (PSA) levels correlating the patient’s race, age, and prostate volume (Wu et al., 2017). Since the use of PSA is limited and controversial, the search for novel PCa-specific biomarkers, especially from non-invasive bio-fluids, is an important task (Wu et al., 2017). Furthermore, while it is assumed that the impacts of the discovered SNPs on tumor initiation and progression cannot be established due to small sample size, we found that certain genes have a vivid association with the expression or tumor type in general agreement. For example, CYP1B1 is known for increases in high-grade PCa and correlates with Gleason grades (Chang et al., 2017), P53 and PRLR are shown to correlate with Gleason grades (Jacobson et al., 2011; Martinez-Gonzalez et al., 2018), MRE11 is known to have an elevated expression associated with progression and poor outcome in PCa (Wang et al., 2019), and ANG is up-regulated in the prostate and involved in prostate intraepithelial neoplasia (PIN) formation (Li et al., 2011; Vanli and Guo-Fu, 2015). As no single biomarker is available for diagnostic and prognostic use (Velonas et al., 2013), the protein–protein interactions complementing our analyses show characteristic associations with a large number of pathways enriched between them (Figure 2B). Although only 5.42% of the protein interaction map density constitutes the pathways, COL6A1, which is known to be upregulated in castration-resistant prostate cancer (CRPC), remains to be the most critical oncogene regulating the androgen signaling pathways (Zhu et al., 2015). This is also in agreement with the pathway association of its sibling genes COL6A2 and COL6A3 (as indicated in light blue edges). Taken together, our results suggest the importance of these pathways and support the potential use of COL6A family members in PCa.

In our PCa cohort, TP53 is the highest prevalent mutation which is in agreement with the previous studies reported (Ecke et al., 2010). Apart from well-known and promising cancer genes, our study uncovered several genes with poorly characterized functional roles in cancer which needs further experimental investigation. For example, *KRIT1* is an intracellular protein with ankyrin repeats and a FERM domain that interacts with the Ras-family GTPase Krev1/Rap1a inferring a role in GTPase signaling cascades. Most of the studies linked were its involvement in cardiovascular development but it is known to be ubiquitously expressed in many cells and tissues (Laberge-le Couteulx et al., 1999). *USH2A*, a gene responsible for Usher syndrome, has been found to be mutated in our cohort demonstrating its role as tumor suppressor (Van Wijk et al., 2004). Although, some other genes such as MCC1, COL6A1, and CYP11B2 are associated with different cancers such as pancreatic, colorectal, and hepatocellular carcinoma, their involvement in PCa is not yet explored (Fukuyama et al., 2008; Fan et al., 2016; Owusu-Ansah et al., 2019). Previous studies, however, have reported the role of diet in PCa (Labbé et al., 2015), so it will be enticing to explore if the genes mentioned above have any role in diet induced PCa. One of the genes, *viz., OPA1*, *is* mutated in our cohort that is important for maintaining normal mitochondrial morphology and function, with deficiency of *OPA1* checking diet-induced obesity and insulin resistance (Pereira et al., 2018). Likewise, *CYP1B1* prevents diet-induced obesity and glucose intolerance through AMPK activation (Liu et al., 2015). We also observed that CTNS (17p13), a gene that causes cystinosis, is associated with multisystem genetic disorder, wherein cysteine is accumulated in different tissues including the adipose and prostate. Strikingly, it is known that vitamin D repletion could be associated with CTNS knockout or mutated mice indicating vitamin D as a key-player in the pathogenesis of PCa (Cheung et al., 2019).

## Conclusion

Prostate cancer is burgeoning in India. Our comprehensive bioinformatics analysis confirmed some characteristic known or unknown mutations from a WES study native to India. Although our study shows characteristic mutations in certain genes, an assay comprising multiple biomarkers that are differentially expressed could be attempted in the future. If this is successful, the number of biomarkers developed will depend on their validation in a large cohort of patients and the translation of these findings to clinical practice. This pilot study, we believe, is decisive to understand the inherent genes and mutations responsible for PCa in India. Furthermore, an attempt was made to develop a conceptual framework for research particularly in propagating information on the causal genes and mutations responsible for PCa. Although the work was limited to a small number of samples studied, we deem this pilot work would have an impending role in understanding mutations that are of particular interest to Indian genealogy.

## Data Availability Statement

The raw reads of WES are deposited in NCBI’s sequence read archive (SRA) with accession PRJNA616165.

## Ethics Statement

The studies involving human participants were reviewed and approved by CK Birla/Rukmani Birla Hospital. The patients/participants provided their written informed consent to participate in this study.

## Author Contributions

AG, MN, and PS analyzed the PCA samples using our NGS pipeline. NS, SG, and BM cross-checked the downstream analysis. MV and DS provided the clinical samples. AM, JB, and NL helped in the Sequenom analysis. AG wrote the first draft with PS. PS proofread the manuscript before uploading and all others agreed to the changes in the manuscript. All authors contributed to the article and approved the submitted version.

## Conflict of Interest

AM was employed by company DNA Xperts Pvt Ltd. The remaining authors declare that the research was conducted in the absence of any commercial or financial relationships that could be construed as a potential conflict of interest.
